# Study of solidification pathway of a MoSiBTiC alloy by optical thermal analysis and *in-situ* observation with electromagnetic levitation

**DOI:** 10.1038/s41598-019-50945-z

**Published:** 2019-10-21

**Authors:** Hiroyuki Fukuyama, Ryogo Sawada, Haruki Nakashima, Makoto Ohtsuka, Kyosuke Yoshimi

**Affiliations:** 1 Institute of Multidisciplinary Research for Advanced Materials, Tohoku University, Sendai, 980-8577 Japan; 2 Department of Materials Science, Graduate School of Engineering, Tohoku University, Sendai, 980-8579 Japan

**Keywords:** Materials science, Structural materials, Techniques and instrumentation

## Abstract

MoSiBTiC alloys are promising candidates for next-generation ultrahigh-temperature materials. However, the phase diagram of these alloys has been unknown. We have developed an ultrahigh-temperature thermal analyser based on blackbody radiation that can be used to analyse the melting and solidification of the alloy 67.5Mo–5Si–10B–8.75Ti–8.75 C (mol%). Furthermore, electromagnetic levitation (EML) was used for *in-situ* observation of solidification and microstructural study of the alloy. On the basis of the results, the following solidification pathway is proposed: Mo solid solution (Mo_ss_) begins to crystallize out as a primary phase at 1955 °C (2228 K) from a liquid state, which is followed by a (Mo_ss_+TiC) eutectic reaction starting at 1900 °C (2173 K). Molybdenum boride (Mo_2_B) phase precipitates from the liquid after the eutectic reaction; however, the Mo_2_B phase may react with the remaining liquid to form Mo_ss_ and Mo_5_SiB_2_ (T_2_) as solidification proceeds. In addition, T_2_ also precipitates as a single phase from the liquid. The remaining liquid reaches the (Mo_ss_ + T_2_ + TiC) ternary eutectic point at 1880 °C (2153 K), and the (Mo_ss_ + T_2_ + Mo_2_C) eutectic reaction finally occurs at 1720 °C (1993 K). This completes the solidification of the MoSiBTiC alloy.

## Introduction

Casting is very important for heat-resistant metallic materials such as creep-resistant steels and Ni-based superalloys. This is because they contain many solute elements that strengthen them, and preventing their segregation during solidification ensures their microstructural homogeneity and longer stability at operation temperature. Because improving heat resistance of creep-resistance steels and Ni-based superalloys improves heat engine efficiency, developing a casting process using phase diagrams is essential for controlling their solidification.

MoSiB-based alloys are promising candidates for next-generation ultrahigh-temperature materials because of their excellent high-temperature strength^1–9^. However, it has been difficult to use them in practical applications because their densities are higher than those of Ni-based superalloys^10^ and their room-temperature fracture toughness is poor^3,11–14^. Recently, Yoshimi *et al*. discovered the major advantage of adding TiC to Mo-Si-B alloys produced by casting^10,15^. The resultant alloys have densities reduced to less than 9 g/cm^3^, excellent high-temperature strength^16^, and good room-temperature toughness^17^. Their cast microstructures are basically composed of Mo solid solution (Mo_ss_), D8_*l*_-structured Mo_5_SiB_2_ (T_2_) and NaCl-type (Ti, Mo)C plus a small amount of MoC_0.5_-type orthorhombic (Mo, Ti)_2_C^18^ depending on composition. It was previously reported^10,15^ that the microstructures are formed through several solidification steps involving three-phase and/or four-phase eutectic reactions. However, the phase diagram of the MoSiBTiC alloy is still unknown.

The phase diagram of the Mo-Si-B ternary system was first constructed by Nowotny *et al*.^19^ in the late 1950s. Almost 40 years later, Nunes *et al*.^20^ reconsidered the liquidus projection for the Mo-rich portion of the Mo-Si-B ternary system on the basis of arc-cast alloy microstructures characterised by scanning electron microscopy (SEM), transmission electron microscopy (TEM) and X-ray diffraction (XRD). Katrych *et al*.^21^ identified the solidification routes, a liquidus projection and a few vertical sections of the ternary phase diagram via differential thermal analysis (DTA), SEM and electron probe microanalysis (EPMA). Yang and Chang^22^ calculated the phase stability, liquidus projection and solidification paths using thermodynamic modelling. Ha *et al*.^23^ modified the primary phase regions in the liquidus projection for the Mo-rich portion using SEM and TEM results. Thus, combining thermal and microstructural analyses is essential to establishing phase diagrams.

However, conventional DTA or differential scanning calorimetry (DSC) is insufficient for studying the phase diagram of a MoSiBTiC alloy because of its more complicated solidification steps and technical problems caused by ultrahigh temperature (>1900 °C). Platinum, usually used as a container and thermocouple material, has a too low melting point (1768 °C) to be used for thermal analysis of MoSiBTiC alloys. In addition, uncertainty in temperature measurement becomes significant at ultrahigh temperatures owing to a lack of well-defined fixed-temperature points.

To overcome these experimental difficulties, we have developed a method for ultrahigh-temperature thermal analysis of MoSiBTiC alloys using blackbody radiation. This involves using a radiation pyrometer to measure blackbody radiation emitted from a sample holder having a blackbody cavity. The radiation pyrometer is calibrated at 1950 °C using the eutectic temperature of the Ru-C system. We use the method to thermally analyse one of the alloys to study the reaction temperatures during its solidification process. Although differential thermal analysers can be operated above 2000 °C using a commercially available W-Re thermocouple, the thermocouple will suffer from drift owing to aging and contamination. The advantage of the new method is in the higher accuracy of the temperature measurement and the larger sample volume, which allows for the detection of smaller heat effects.

The thermal analysis requires a way to relate reaction heat peaks to corresponding microstructure formation to reveal the solidification pathway of the MoSiBTiC alloy. Here, we use EML to *in-situ* observe its solidification. We developed an EML technique coupled with a static magnetic field in our previous work^24–28^. This technique enables high-precision, non-contact thermophysical measurements of high-temperature melts. In this work, we use the technique to *in-situ* observe the solidification of the MoSiBTiC melt, and study the microstructure obtained from a quenched sample of the EML experiment. Finally, the solidification of the alloy is discussed in light of the thermal analysis and *in-situ* observation.

## Principle of Ultrahigh-Temperature Thermal Analysis

**Figure 1:**
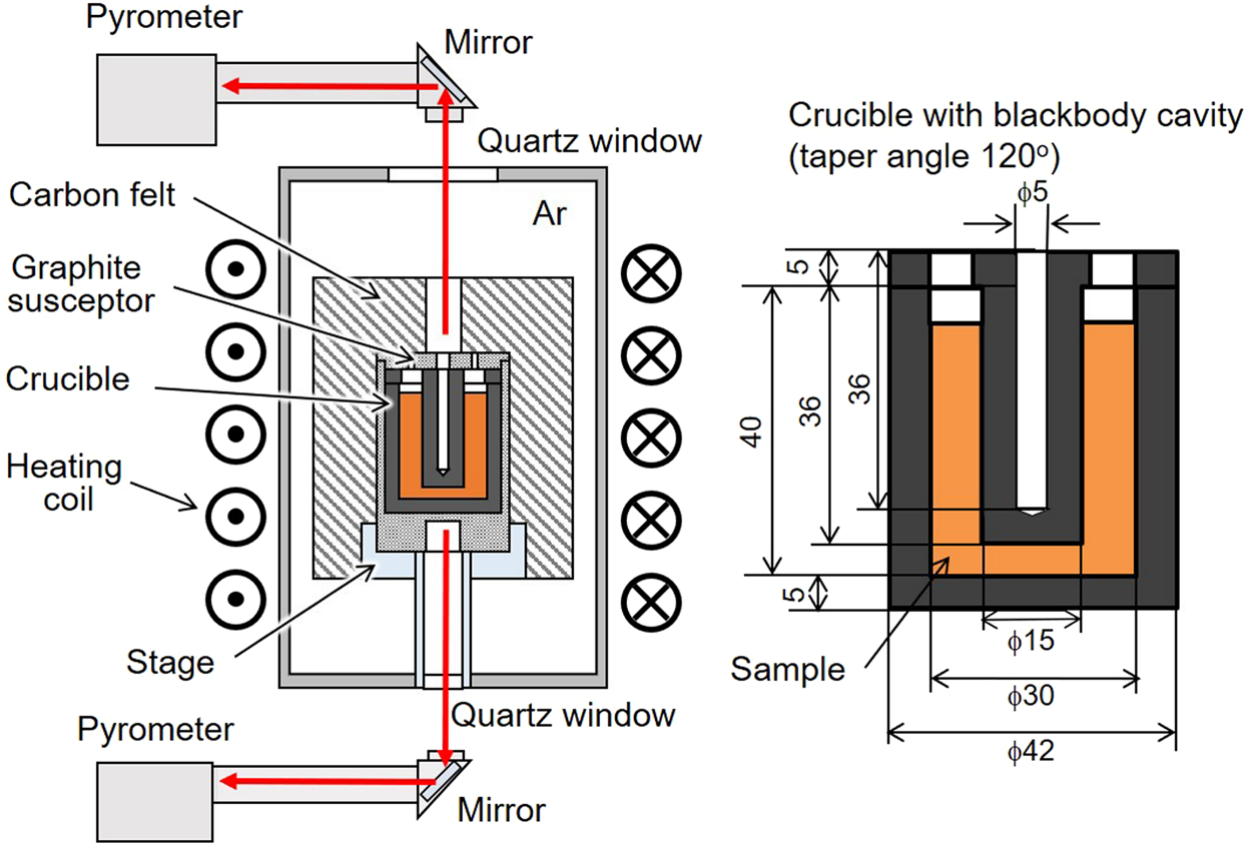
Left: schematic of the ultra-high-temperature thermal-analysis apparatus; Right: crucible with a blackbody cavity.

Figure 1 is a schematic of our ultrahigh-temperature thermal analysis apparatus. This apparatus consists of a radio-frequency furnace, two pyrometers and a crucible having a blackbody cavity. A sample is filled into the crucible and heated through a graphite susceptor by applying a radio-frequency current to the heating coil. The radiation from the blackbody cavity is observed with a pyrometer at the top of the apparatus through a quartz glass and a mirror, measuring the sample temperature *T*_s_. The susceptor temperature *T*_c_ is monitored with a pyrometer at the bottom of the apparatus, which is used for temperature control. The temperature determination of the sample is based on the Planck equation

1 $${E}_{{\rm{b}}}=\frac{{C}_{1}}{{\lambda }^{5}\{\exp (\frac{{C}_{2}}{\lambda T})-1\}}\,,$$

where *E*_b_ is the emissive blackbody power, *λ* is the wavelength, *T* is the absolute temperature, and *C*_1_ and *C*_2_ are the first and second radiation constants equal to 3.7415 × 10^−16^ W·m^2^ and 1.4388 × 10^−2^ m·K, respectively. The emissivity *ε*_c_ of the blackbody cavity is expressed in terms of the emissivity ε_m_ of the crucible material and aspect ratio $\alpha$ of the cavity^29^:

2 $${\varepsilon }_{{\rm{c}}}=1-\frac{(1-{\varepsilon }_{{\rm{m}}})\pi }{{\alpha }^{2}}\,.$$

The value of *ε*_c_ was calculated as 0.999 for graphite with the aspect ratio of the blackbody cavity (diameter of 5 mm; depth of 36 mm). The emissive power of the blackbody cavity, *E*_c_, is

3 $${E}_{{\rm{c}}}=\tau {\varepsilon }_{{\rm{c}}}{E}_{{\rm{b}}}={\varepsilon }_{{\rm{app}}}{E}_{{\rm{b}}}\,,$$

where *τ* is the absorptivity of the quartz glass and mirror, and *ε*_app_ is the apparent emissivity. From Eqs (1) and (3), the absolute temperature is expressed using the emissive power of the blackbody cavity, *E*_c_:

4 $$T=\frac{{C}_{2}}{\lambda \{{\rm{l}}{\rm{n}}(\frac{{\varepsilon }_{{\rm{a}}{\rm{p}}{\rm{p}}}{C}_{1}}{{E}_{{\rm{c}}}{\lambda }^{5}}+1)\}}\,.$$

In this study, *ε*_app_ was obtained using fixed-temperature points. The highest fixed-temperature point defined by the international temperature scale (ITS–90) is the melting point *T*_m_ of Cu (1084.62 °C)^30^. Yamada *et al*.^31^ have proposed eutectic temperatures (*T*_E_) of metal-carbon binary systems for fixed-temperature points above *T*_m_ for Cu. Because the liquidus temperature of MoSiBTiC alloys seems to be higher than 1900 °C, *T*_E_ values for Ru–C (1953 °C^31^) and Ni–C (1329 °C^31^) alloys were chosen as the fixed points in addition to *T*_m_ for Cu. We considered the effect of eutectic reaction kinetics on measuring fixed points on the basis of the Fe–C eutectic transition temperature reported by Sasajima *et al*.^32^

## Results

### Ultrahigh-temperature thermal analysis

#### Fixed-temperature points

**Figure 2:**
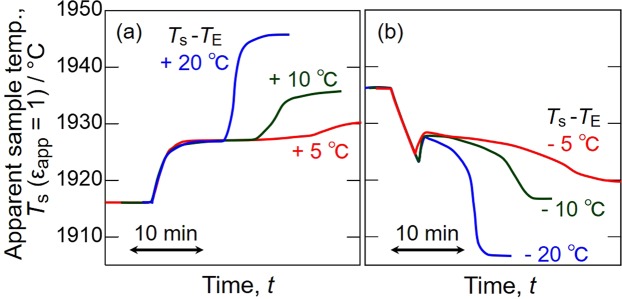
Apparent temperature curves of Ru-C alloy on (**a**) heating and (**b**) cooling.

**Figure 3:**
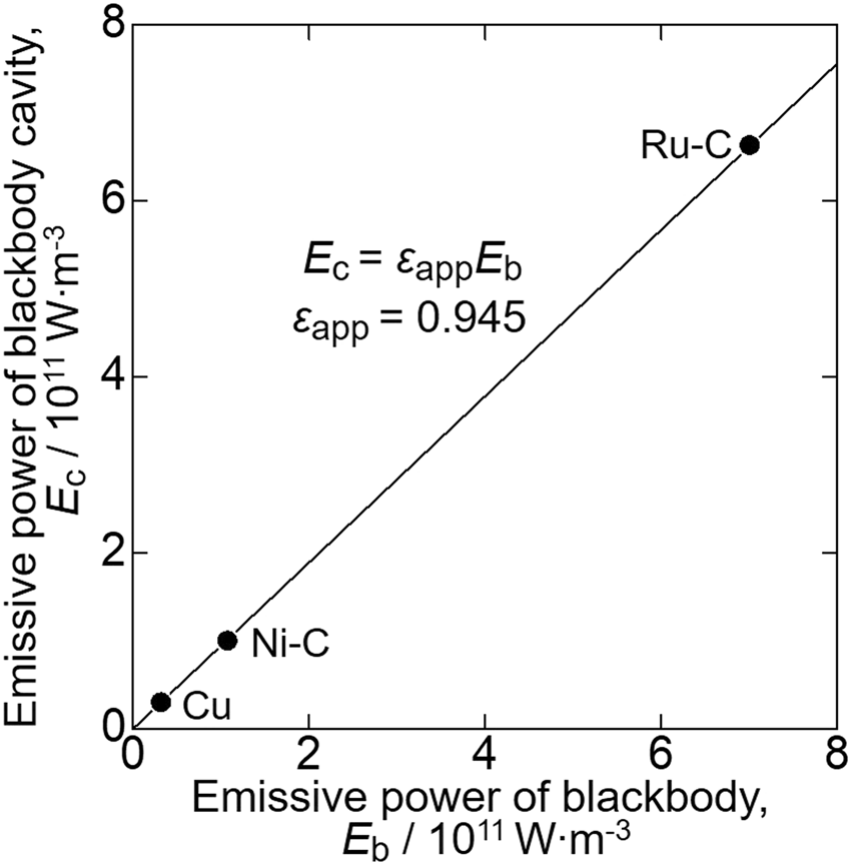
Relationship between *E*_b_ and *E*_c_ at *T*_m_ for Cu and *T*_E_ for Ni-C and Ru-C alloys.

The ultrahigh-temperature analyser was calibrated with the melting point of pure Cu and the eutectic temperatures of the Ru–C and Ni–C alloys. As an example, Fig. 2 shows the apparent temperature curves of the Ru–C eutectic alloy obtained upon heating and cooling assuming *ε*_app_ = 1. The sample temperature was initially set lower than *T*_E_ by 10 °C and kept there for thermal homogenisation. The sample was heated again to a temperature higher than *T*_E_ by 5, 10 and 20 °C until the eutectic plateau was completed as shown in Fig. 2(a). After the eutectic melting plateau was completed, the sample was cooled to a temperature lower than *T*_E_ by 5, 10 and 20 °C to obtain the eutectic plateau on cooling (Fig. 2(b)). In Fig. 2(a), the duration of the eutectic plateau on heating decreases, meaning the eutectic reaction rate increases, with increasing set temperature. However, the eutectic temperature does not change with reaction rate. The cooling curves in Fig. 2(b) show that the eutectic temperature after undercooling slightly depends on set temperature. This difference is understood in terms of eutectic solidification kinetics. Thus, the eutectic plateau obtained on heating was used as the fixed point in this study, and its average apparent *T*_E_ for the Ru-C alloy was 1928 °C. By substituting the apparent *T*_E_ and *ε*_app_ = 1 into Eq. (4), *E*_c_ was obtained for the Ru–C alloy, and *E*_b_ was estimated via Eq. (1) for the true *T*_E_ of the Ru–C alloy (1953 °C). Similar measurements were conducted to obtain *T*_E_ for the Ni–C alloy and *T*_m_ for Cu. Figure 3 shows a linear relationship between *E*_b_ and *E*_c_ for these fixed-temperature points. The apparent emissivity *ε*_app_ was determined to be 0.945 from the slope. From these results, the uncertainty in temperature measurements for the thermal analysis was evaluated as ±0.4% within a temperature range from 1084.62 to 1953 °C.

#### MoSiBTiC alloy

**Figure 4:**
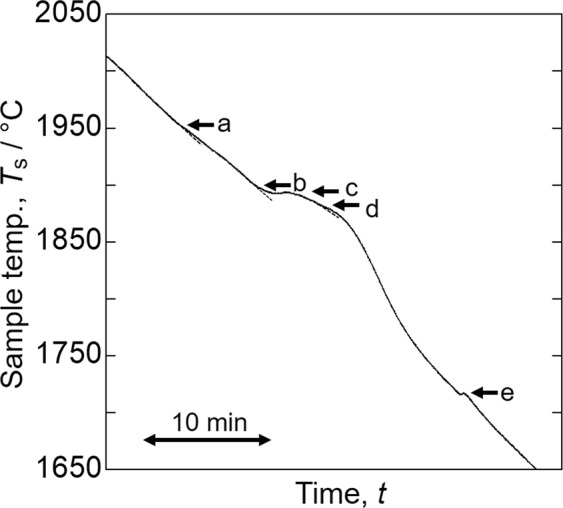
Cooling curve of the MoSiBTiC alloy.

The composition of the MoSiBTiC alloy used in this study was 67.5Mo–5Si–10B–8.75Ti–8.75 C (mol%). The alloy was thermally analysed using the above method with *ε*_app_ = 0.945. Heating and cooling steps were repeated three times. Figure 4 shows a typical cooling curve of the MoSiBTiC alloy obtained in the second cooling step. Five inflection points appear on the cooling curve. Table 1 shows the temperatures of all inflection points and their averages for three cooling curves. Reactions corresponding to the inflection points will be discussed later.

**Table 1:** Inflection point temperatures of MoSiBTiC alloy (67.5Mo–5Si–10B–8.75Ti–8.75 C in mol%).

Inflection points	1^st^ (°C)	2^nd^ (°C)	3^rd^ (°C)	Average (°C)
A	1950	1954	1962	1955 (2228 K)
B	1899	1903	1899	1900 (2173 K)
C	1894	1894	1894	1894 (2167 K)
D	—	1881	1879	1880 (2153 K)
E	1719	1717	1725	1720 (1993 K)

### Solidification behavior of MoSiBTiC melt obtained by EML

#### Multiple recalescence

**Figure 5:**
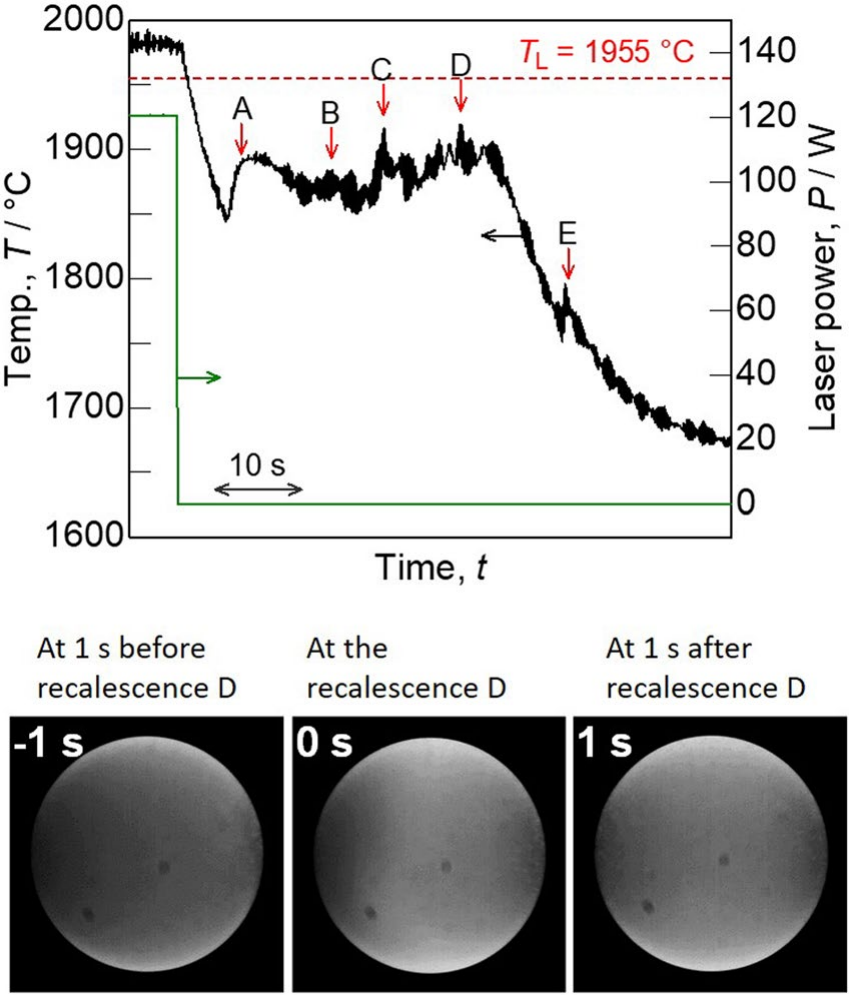
Top: temperature profile of the MoSiBTiC alloy melt levitated via EML on cooling. Multiple recalescences (A–E) were recorded. Bottom: sequential top-view image of the alloy melt shows light emission caused by recalescence D.

The MoSiBTiC alloy was levitated and melted using EML in a static magnetic field to observe the solidification of the melt. Figure 5 (top) shows the cooling temperature profile of the MoSiBTiC alloy melt after the heating laser was turned off. We recorded multiple light emissions from the alloy melt owing to the release of latent heat (recalescence), and these emissions were assigned to peaks A–E on the temperature profile from the *in-situ* CCD camera observation. An example is the bottom of Fig. 5, which is a sequential top-view image of the alloy melt around recalescence D, at which the melt surface became brighter. Consequently, solidification of the melt started with recalescence A and finished with recalescence E. Here, the sample temperature was measured using a pyrometer calibrated with the liquidus temperature (1955 °C) of the alloy as determined in the preceding section. However, after recalescence, the sample temperature was affected by solids precipitated on the melt surface because the partially solidified portion had a different emissivity from that of the melt. For this reason, the recalescence points A–E correspond to the inflection points a–e in Fig. 4, but the recalescence temperatures do not agree with the inflection points.

#### Solidification microstructural analysis

**Figure 6:**
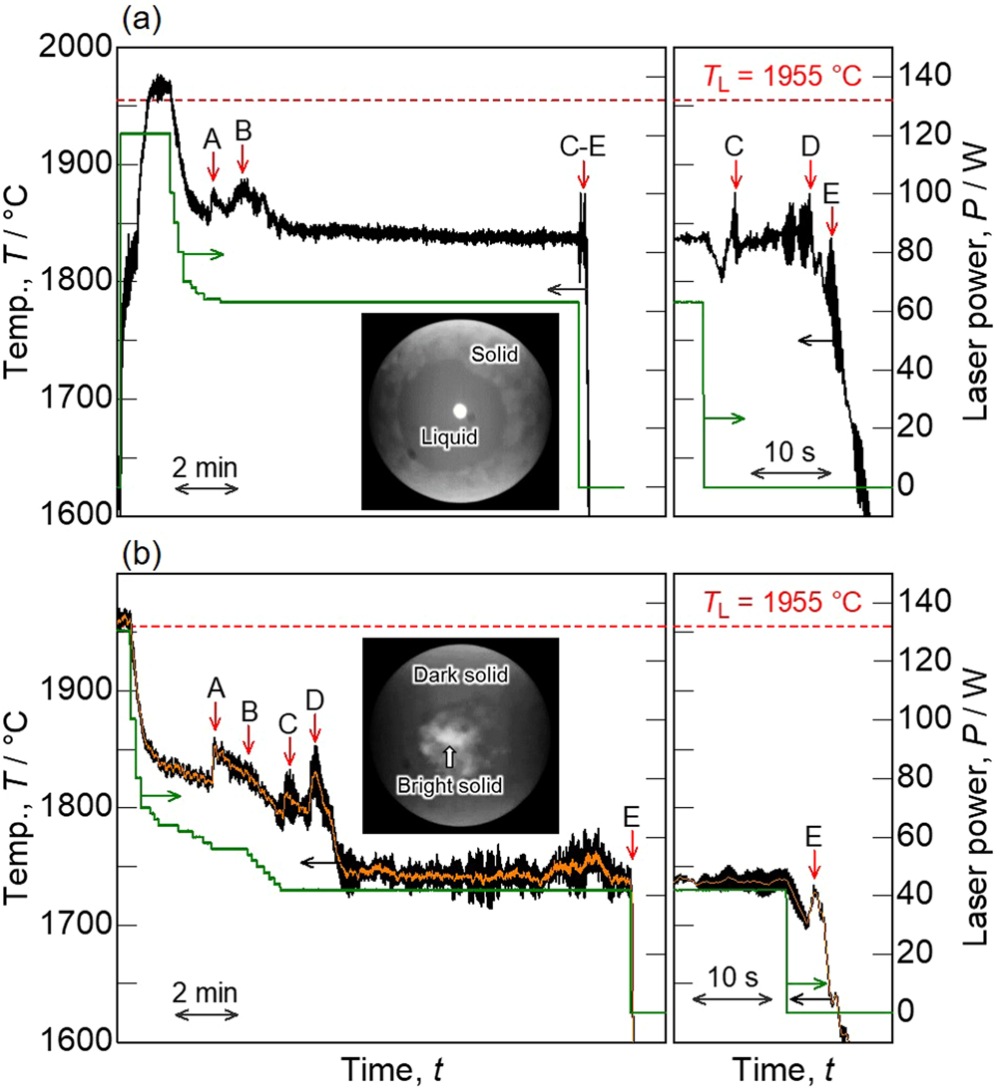
Temperature profile of the MoSiBTiC alloy melt levitated via EML on cooling. (**a**) The alloy was held for 10 min at the temperature after recalescence B, and (**b**) the alloy was held for 10 min after recalescence D. Partial temperature profiles with an enlarged time scale are also added to show peaks during the rapid cooling. The inserts in both figures are top-view images of the partially solidified alloy during the holding time.

The levitated MoSiBTiC alloy melt was rapidly solidified from recalescence in EML to freeze the microstructure at that temperature. For microstructural analysis, the partially solidified alloys were held at these temperatures for 10 min just after recalescences B and D as shown in Fig. 6(a,b), respectively, to develop the microstructures. This was followed by the rapid solidification under a He gas flow. This figure also shows partial temperature profiles during the rapid cooling with an enlarged time scale, and top-view images of the partially solidified alloys during the holding time. Solid precipitated from the alloy melt is seen in the laser spot at the centre. Liquid and solid phases are distinguished by their emissivity difference on the alloy surface.

**Figure 7:**
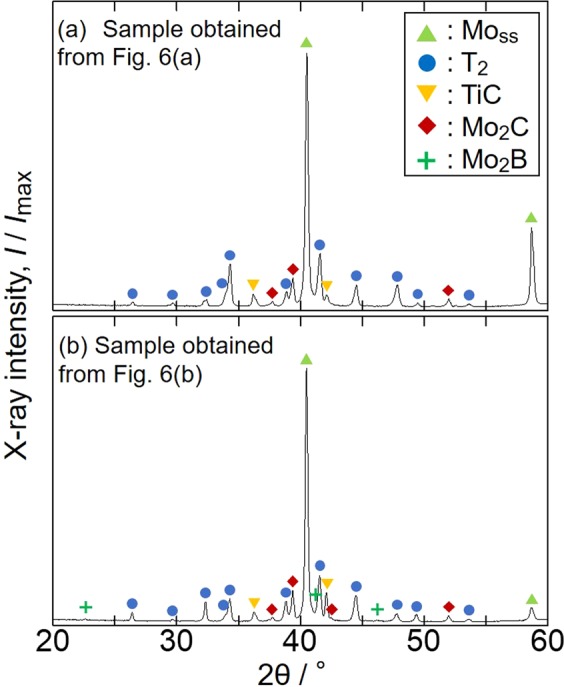
XRD profiles of the rapidly solidified MoSiBTiC alloys obtained from the experiments of Fig. 6(a,b).

Figure 7(a,b) show the XRD profiles of the quenched samples obtained via the EML experiments of Fig. 6(a,b), respectively. The diffraction peaks of molybdenum solid solution (Mo_ss_) were clearly observed, and Mo_5_SiB_2_ (T_2_), TiC, Mo_2_C and Mo_2_B phases were also identified in the quenched samples.

**Figure 8:**
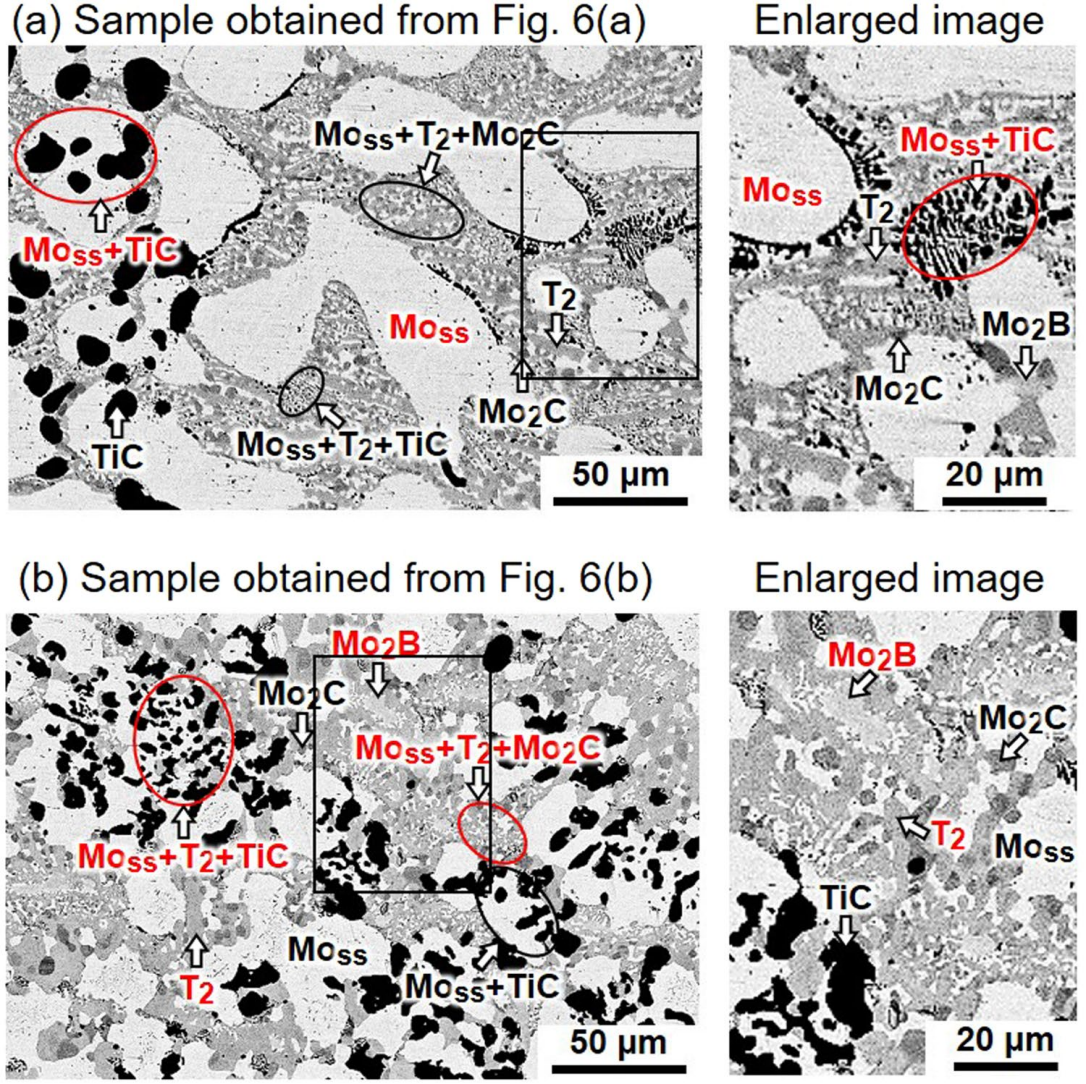
Cross-sectional SEM images of rapidly solidified MoSiBTiC alloys obtained from the experiments of Fig. 6(a,b).

Figure 8(a,b) show the cross-sectional SEM images of the quenched samples obtained via the EML experiments of Fig. 6(a,b), respectively. Phase identification was performed with SEM energy-dispersive X-ray spectrometry (SEM-EDX) considering the XRD results. As shown in Fig. 8(a), the microstructure has large Mo_ss_ grains (white), which should be primarily crystalized in the alloy melt. A TiC (black) phase is seen in the (Mo_ss_ + TiC) binary-eutectic structure, as are some minor phases: Mo_2_B (light grey), T_2_ (grey) and Mo_2_C (dark grey). Furthermore, fine (Mo_ss_ + T_2_ + TiC) and (Mo_ss_ + T_2_ + Mo_2_C) ternary-eutectic structures are observed. In the microstructure in Fig. 8(b), Mo_ss_ and T_2_ phases and (Mo_ss_ + TiC) binary-eutectic structure are seen. The (Mo_ss_ + T_2_ + TiC) ternary-eutectic structure is more developed than the structure in Fig. 8(a). A fine (Mo_ss_ + T_2_ + Mo_2_C) ternary-eutectic structure is also observed.

## Discussion

### Solidification pathway

We discuss the solidification pathway of the MoSiBTiC alloy (67.5Mo–5Si–10B–8.75Ti–8.75 C in mol%) in light of the thermal analysis and EML experiments. The results are as follows:

1. Five inflection points (a–e) were detected on the cooling curve in Fig. 4.
2. Five recalescences (A–E) were *in-situ* observed as shown in Fig. 5.
3. Mo_ss_, Mo_2_B and T_2_ single phases, one binary-eutectic structure (Mo_ss_ + TiC) and two ternary-eutectic structures (Mo_ss_ + T_2_ + TiC) and (Mo_ss_ + T_2_ + Mo_2_C) were observed in the microstructures of Fig. 8(a,b).

From the above results, we propose the solidification pathway for the MoSiBTiC alloy in Table 2. Inflection points a–e in Fig. 4 correspond to recalescences A–E in Fig. 5, respectively. We note again that, except for the liquidus temperature, the recalescence temperatures in Fig. 5 do not agree with the inflection points of Fig. 4 because the emissivity of the MoSiBTiC alloy melt levitated by EML was altered by solids crystalized on the melt surface during cooling.

**Table 2:** Solidification pathway of MoSiBTiC alloy (67.5Mo–5Si–10B–8.75Ti–8.75 C in mol%).

Inflection point obtained in Fig. 4	Recalescence appeared in Fig. 5	Reactions occurred at the inflection temperature
a: 1955 °C (2228 K)	A	Liq. → Mo_ss_ primary
b: 1900 °C (2173 K)	B	Liq. → Mo_ss_ + TiC (eutectic)
c: 1894 °C (2167 K)	C	Liq. → Mo_2_B Liq. + Mo_2_B → Mo_ss_ + T_2_
d: 1880 °C (2153 K)	D	Liq. → T_2_ Liq. → Mo_ss_ + T_2_ + TiC (eutectic)
e: 1720 °C (1993 K)	E	Liq. → Mo_ss_ + T_2_ + Mo_2_C (eutectic)

#### Effect of undercooling on the solidification pathway

Deep undercooling may affect the solidification pathway of alloy melts. For example, Lü and Wang^33,34^ reported that the transition of the primary phase crystallization to the non-equilibrium peritectic phase crystallization is dependent on the degree of undercooling of the melt. In the present study, the undercooling of the MoSiBTiC alloy melt reached 100–130 °C as shown in Figs 5 and 6. After the first undercooling, recalescence A occurred but the sample temperature did not increase to the initial liquidus temperature. This does not imply a metastable phase formation, but was simply caused by the liquidus composition shift toward the boundary line between Mo_ss_ and TiC primary phases during recalescence A and the subsequent (Mo_ss_ + TiC) binary-eutectic reaction. Therefore, the sample temperature rise was lowered from the initial liquidus temperature and was limited to the (Mo_ss_ + TiC) binary-eutectic temperature (1900 °C). This is understood by the microstructural observation. The microstructures shown in Fig. 8 indicate that the large Mo_ss_ grains were primarily crystalized in the alloy melt, which was followed by the (Mo_ss_ + TiC) binary-eutectic reaction. Thus, the undercooling did not affect the solidification pathway in the present study.

#### Discussion based on the Mo-Si-B-TiC phase diagram

**Figure 9:**
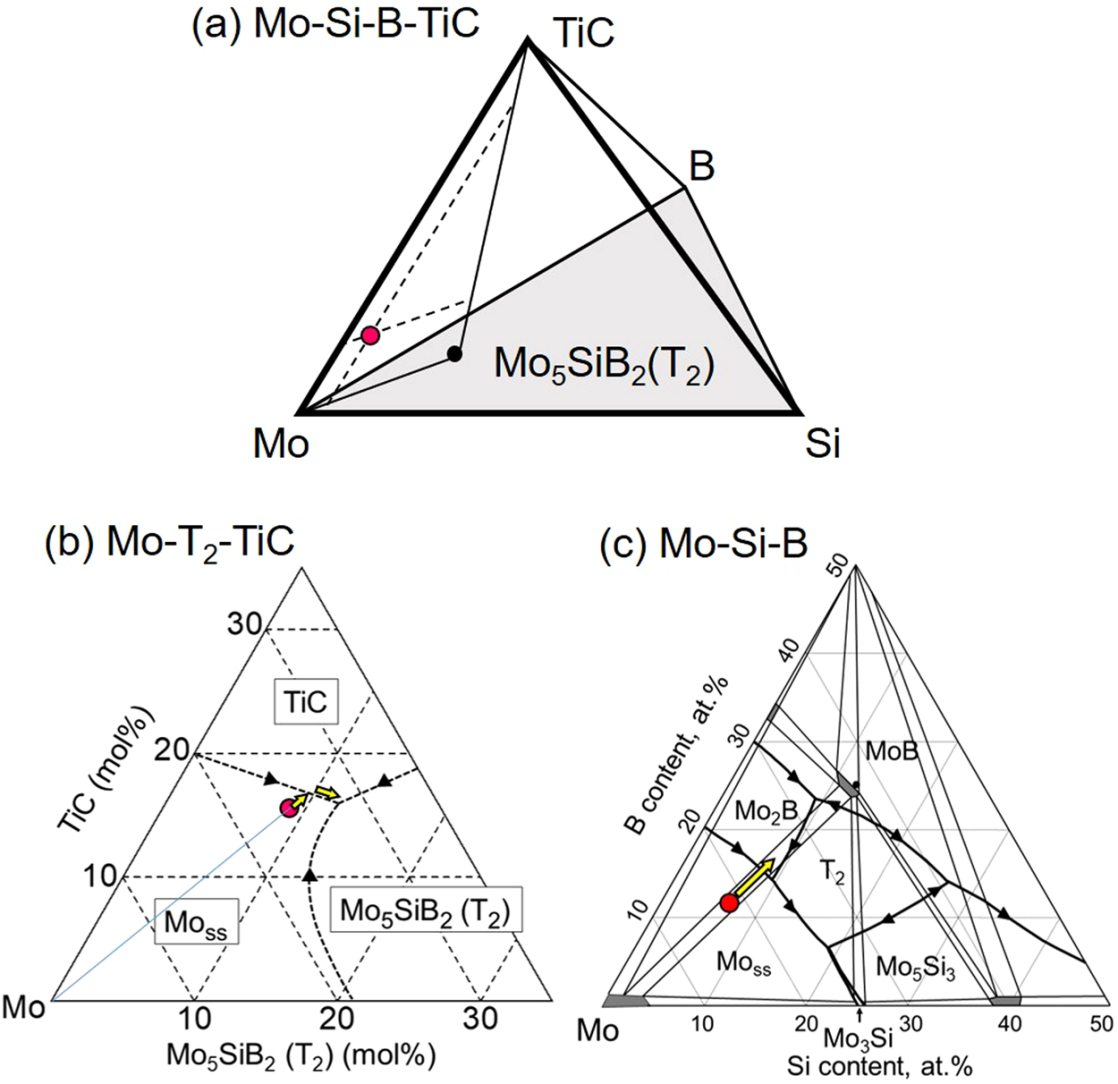
(**a**) Mo-Si-B-TiC quaternary phase diagram, (**b**) Mo-Mo_5_SiB_2_(T_2_)-TiC ternary phase diagram and (**c**) Mo-Si-B ternary phase diagram^23^.

The chemical composition of the MoSiBTiC alloy (67.5Mo–5Si–10B–8.75Ti–8.75 C in mol%) we used is located on the Mo-Si-B-TiC quaternary phase diagram in Fig. 9(a). The alloy composition is also expressed as 75.6Mo–8.9Mo_5_SiB_2_ (T_2_)–15.6TiC in mol% in the Mo–T_2_–TiC ternary system, and is plotted in Fig. 9(b). In this ternary system, the alloy composition is initially located in the Mo_ss_ primary phase region. Therefore, after the Mo_ss_ primary phase precipitates at 1955 °C (inflection point a), the liquid composition shifts toward the Mo_ss_/TiC boundary lines, then the binary eutectic reaction

5 $${\rm{Liq}}.\to {{\rm{Mo}}}_{{\rm{ss}}}+{\rm{TiC}}$$

occurs at 1900 °C (inflection point b) in Fig. 9(b). Eremenko and Velikanova^35^ reported a eutectic temperature of 2175 °C for the Mo–TiC binary system. Here, the reaction (5) occurred at 1900 °C in the Mo–Si–B–TiC system.

However, the alloy composition is projected onto the basal plane (the Mo–Si–B ternary system) as shown in Fig. 9(c)^23^. The initial alloy composition exists in the Mo_ss_ primary phase region on the connecting line between Mo and Mo_5_SiB_2_ (T_2_). However, Mo_ss_ content in the liquid is decreased by the eutectic reaction (5), and therefore, the liquid composition moves into the Mo_2_B primary phase region as shown in Fig. 9(c). However, this Mo_2_B phase soon reacts with the liquid to form Mo_ss_ and T_2_ via the reaction

6 $${\rm{L}}{\rm{i}}{\rm{q}}.+{{\rm{M}}{\rm{o}}}_{2}{\rm{B}}\to {{\rm{M}}{\rm{o}}}_{{\rm{s}}{\rm{s}}}+{{\rm{T}}}_{2}\,.$$

Nunes *et al*.^36^ reported that this reaction occurs in the four-phase equilibrium at about 2100 °C in the Mo–Si–B ternary system. In our study, the reaction (6) occurred at 1894 °C (inflection point c) in the Mo–Si–B–TiC system.

Going back to the Mo–T_2_–TiC ternary phase diagram (Fig. 9(b)), the liquid reaches the ternary eutectic point after the eutectic reaction (5), then the following reaction occurs at 1880 °C (inflection point d):

7 $${\rm{L}}{\rm{i}}{\rm{q}}.\to {{\rm{M}}{\rm{o}}}_{{\rm{s}}{\rm{s}}}+{{\rm{T}}}_{2}+{\rm{T}}{\rm{i}}{\rm{C}}\,.$$

In addition, the elongated shape of the T_2_ grains in Fig. 8(b) indicates that the T_2_ phase precipitated out as a single phase from the liquid phase between reactions (6) and (7).

Finally, the (Mo_ss_ + T_2_ + Mo_2_C) eutectic reaction occurred at 1720 °C (inflection point e), which does not appear on the Mo–Si–B–TiC phase diagram. The order of the two ternary eutectic reactions (6) and (7) is judged from the relatively well-developed (Mo_ss_ + T_2_ + TiC) eutectic structure compared with the (Mo_ss_ + T_2_ + Mo_2_C) eutectic structure. Thus, the solidification is complete.

Miyamoto *et al*.^10^ reported the solidification pathway of a similar MoSiBTiC alloy designated as B_2_ (70.0Mo–3.3Si–6.7B–10.0Ti–10.0 C in mol%): Mo_ss_ (primary)→(Mo_ss_ + TiC) eutectic→T_2_→(Mo_ss_ + T_2_ + TiC) eutectic→(Mo_ss_ + T_2_ + Mo_2_C) eutectic. This process was proposed on the basis of microstructures of as-cast alloy from arc melting and heat-treated alloy at 1800 °C for 24 h. It is difficult to figure out the full solidification process only from microstructural observation. Here, our proposed solidification pathway based on the combination of thermal analysis, *in-situ* observation and EML quenching supports the results obtained by Miyamoto *et al*.

In summary, a method for ultra-high-temperature thermal analysis was developed. The maximum measurement error was 5 °C with a temperature range from 1084.62 to 1953 °C. By using this technique, the MoSiBTiC alloy (67.5Mo–5Si–10B–8.75Ti–8.75 C in mol%) was thermally analysed. EML experiments were also conducted to observe the *in-situ* solidification of the MoSiBTiC alloy melt, and to study the microstructure of the rapidly solidified sample. The solidification pathway was finally proposed as given in Table 2.

## Methods

### Ultra-high-temperature thermal analysis

#### Fixed-temperature points

The raw materials for the Ru–C alloy were Ru (99.9 mass%; particle diameter < 500 µm; JX Nippon Mining & Metals, Japan) and C (99.99 mass%; particle diameter <10 µm; Kojundo Chemical Laboratory, Japan) powders. Ru–C mixed powder with a composition of Ru–11 mol% C was filled into a graphite crucible having a blackbody cavity with a tape angle of 120°, and then heated and cooled in a radio-frequency furnace under an Ar atmosphere at an Ar flow rate of 10 mL/min. The apparent sample temperature *T*_s_ was determined assuming *ε*_app_ = 1 with a top pyrometer measuring the radiation from the blackbody cavity. Initially the Ru-C sample was kept at *T*_s_ = *T*_E_ − 10 °C to stabilise the furnace temperature, after which it was heated to *T*_s_ = *T*_E_ + (5, 10, 20) °C at a rate of 5 °C/min. After the sample was kept at *T*_s_ = *T*_E_ + 10 °C, it was cooled to *T*_s_ = *T*_E_ − (5, 10, 20) °C at a rate of 5 °C/min. This procedure was used for other fixed-point measurements with *T*_m_ for Cu and *T*_E_ for Ni–C. A Cu rod (99.99 mass%; diameter of 2 mm; length of 2 mm; Kojundo Chemical Laboratory, Japan) and Ni rod (99.99 mass%; diameter of 2 mm; length of 2 mm; Rare Metallic, Japan) were used as raw materials. The Ni–C alloy was prepared with the composition Ni–4 mol% C. The apparent emissivity *ε*_app_ of the pyrometer was determined in advance using *T*_m_ for Cu and *T*_E_ for the Ni–C and Ru–C alloys.

#### MoSiBTiC alloy

The MoSiBTiC alloy was prepared by arc-melting from Mo (99.99 mass%; diameter of 10 mm; length of 300 mm; A.L.M.T.), Si (99.999 mass%; particle size of 2–5 mm; Kojundo Chemical Laboratory, Japan), B (99.5 mass%; particle size of 3–7 mm; Kojundo Chemical Laboratory, Japan) and TiC (99 mass%; particle size of 2–5 µm; Kojundo Chemical Laboratory, Japan) as raw materials. The chemical composition of the alloy was 67.5Mo–5Si–10B–8.75Ti–8.75 C (mol%). Here, T_2_ is a ternary compound (Mo_5_SiB_2_). A CaO-stabilised ZrO_2_ crucible having a blackbody cavity was used to hold the MoSiBTiC alloy (about 87 g) instead of a graphite crucible to prevent carbon contamination. The alloy was heated and cooled in a radio-frequency furnace under an Ar atmosphere at an Ar flow rate of 10 mL/min. The sample was heated at a rate of 10 °C/min and kept around 2000 °C for 30 min to ensure homogeneous melt, and subsequently cooled at a rate of 10 °C/min. The sample temperature was determined during heating and cooling with a top pyrometer measuring the radiation from the blackbody cavity. The inflection points on the cooling curves were treated as the phase transformation temperatures of the MoSiBTiC alloy.

### EML experiments on the MoSiBTiC alloy

**Figure 10:**
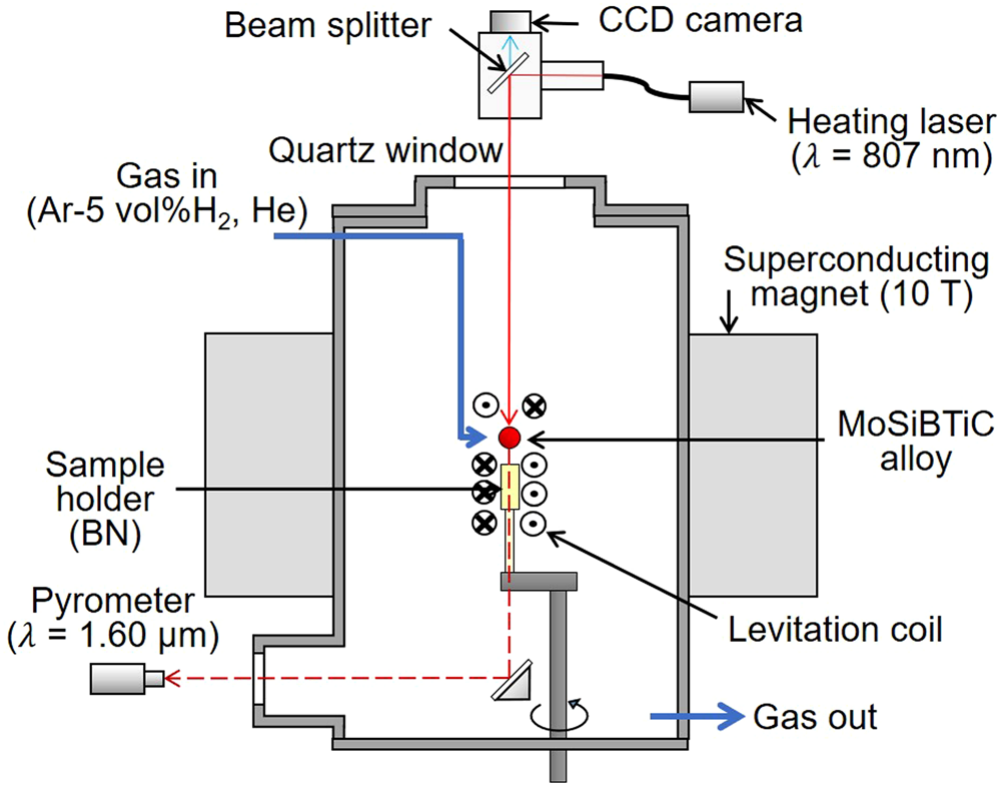
Schematic of the EML apparatus.

The schematic of the EML apparatus is shown in Fig. 10. After the MoSiBTiC sample was placed on the sample holder made of BN, the chamber was evacuated to 10^−2^ Pa using a rotary pump and a turbomolecular pump. The chamber was filled with Ar–5 vol% H_2_ gas after evacuation. Alternating electric current was applied to the levitation coil to levitate and heat the sample. The sample was melted with a heating laser (wavelength of 807 nm; power of 8–140 W; Jenoptik Laserdiode, Japan; NBT-S140mkII), and the sample temperature was controlled with the laser power. Ar–5 vol% H_2_ gas was supplied at a rate of 3 L/min to prevent sample oxidation. A static magnetic field of 10 T was applied to the sample droplet to suppress both its translational motion and convection. For *in-situ* observation of solidification, the sample droplet was filmed with a CCD camera (MC1310; MIKROTRON GmbH, Germany) with a resolution of 512 × 512 pixels at 60 fps. The sample temperature was measured using a single-colour pyrometer (temperature range of 450–2500 °C; spectral range of 1.45–1.8 μm; IGA140/MB25; IMPAC Pyrometers; LumaSense Technologies, Germany), which was calibrated at the liquidus temperature of the MoSiBTiC alloy. The sample was held at the temperature right after recalescence for 10 min to grow crystallized grains. After holding each temperature, the heating laser was turned off and He gas was blown at a rate of 5 L/min to quench the sample. SEM (JEOL; JCM-5700) and XRD (Bruker; D2 Phaser) were used to observe and analyse microstructures in a cross-section of the solidified sample.

## Supplementary Information

Supplementary information
